# Publisher Correction: Genomic analyses of early responses to radiation in glioblastoma reveal new alterations at transcription, splicing, and translation levels

**DOI:** 10.1038/s41598-020-69585-9

**Published:** 2020-08-04

**Authors:** Saket Choudhary, Suzanne C. Burns, Hoda Mirsafian, Wenzheng Li, Dat T. Vo, Mei Qiao, Xiufen Lei, Andrew D. Smith, Luiz O. Penalva

**Affiliations:** 1 Computational Biology and Bioinformatics, University of Southern California, California, USA; 2 Greheey Children’s Research Institute, University of Texas Health Science Center At San Antonio, Texas, USA; 3 Department of Cell Systems and Anatomy, University of Texas Health Science Center At San Antonio, Texas, USA; 4 Department of Radiation Oncology, University of Texas Southwestern Medical Center, Texas, USA

Correction to: *Scientific Reports*, 10.1038/s41598-020-65638-1, published online 02 June 2020

The original version of this Article contained some errors in the title of the paper. The words “in glioblastoma” were incorrectly given as “inglioblastoma”. Additionally, the words “transcription, splicing” were incorrectly given as “transcription,splicing”. This has now been corrected in the PDF and HTML versions of the Article.

