# Supplementary material for: Genomic analyses of early responses to radiation inglioblastoma reveal new alterations at transcription,splicing, and translation levels
Source: Sci Rep. 2020 Jun 2;10:8979. doi: 10.1038/s41598-020-65638-1 (PMC7265345; doi:10.1038/s41598-020-65638-1)
Supplement: Supplementary file 1 — Supplementary Table S1 [file 41598_2020_65638_MOESM1_ESM.pdf]

Supplementary Information: Genomic analyses of early responses to radiation in glioblastoma reveal new alterations at transcription, splicing, and translation levels

Saket Choudhary [*1], Suzanne C. Burns [*†2], Hoda Mirsafian[1], Wenzheng Li[1], Dat T. Vo[4], Mei Qiao[2], Xiufen Lei[2], Andrew D. Smith[1], and Luiz O. Penalva [‡2,3]

[1]Computational Biology and Bioinformatics, University of Southern California
[2]Greheey Children's Research Institute, University of Texas Health Science Center at San Antonio
[3]Department of Cell Systems and Anatomy, University of Texas Health Science Center at San Antonio
[4]Department of Radiation Oncology, University of Texas Southwestern Medical Center

Supplementary Figures

[*]Equal contributor
[†]In memoriam
[‡]Correspondence to: penalva@uthsca.edu

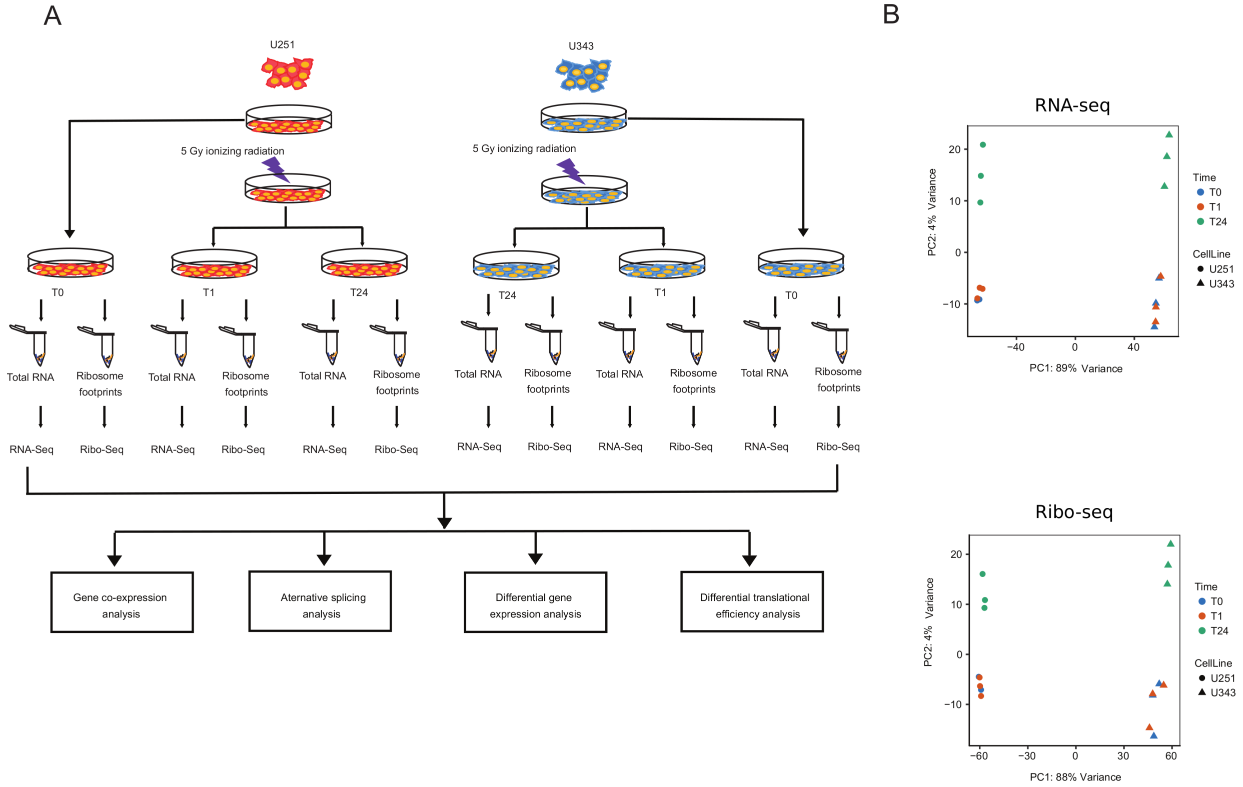

Figure S1: **Experimental design and Principal Component Analysis of RNA-Seq and Ribo-Seq data from glioblastoma cell lines.** A) Schematic representation of experimental protocol followed for radiation exposure of glioma cell lines, and sample preparation for sequencing the RNA and ribosome footprints. B) Principal component analyses performed on normalized log-transformed read counts of RNA-Seq and Ribo-Seq datasets.

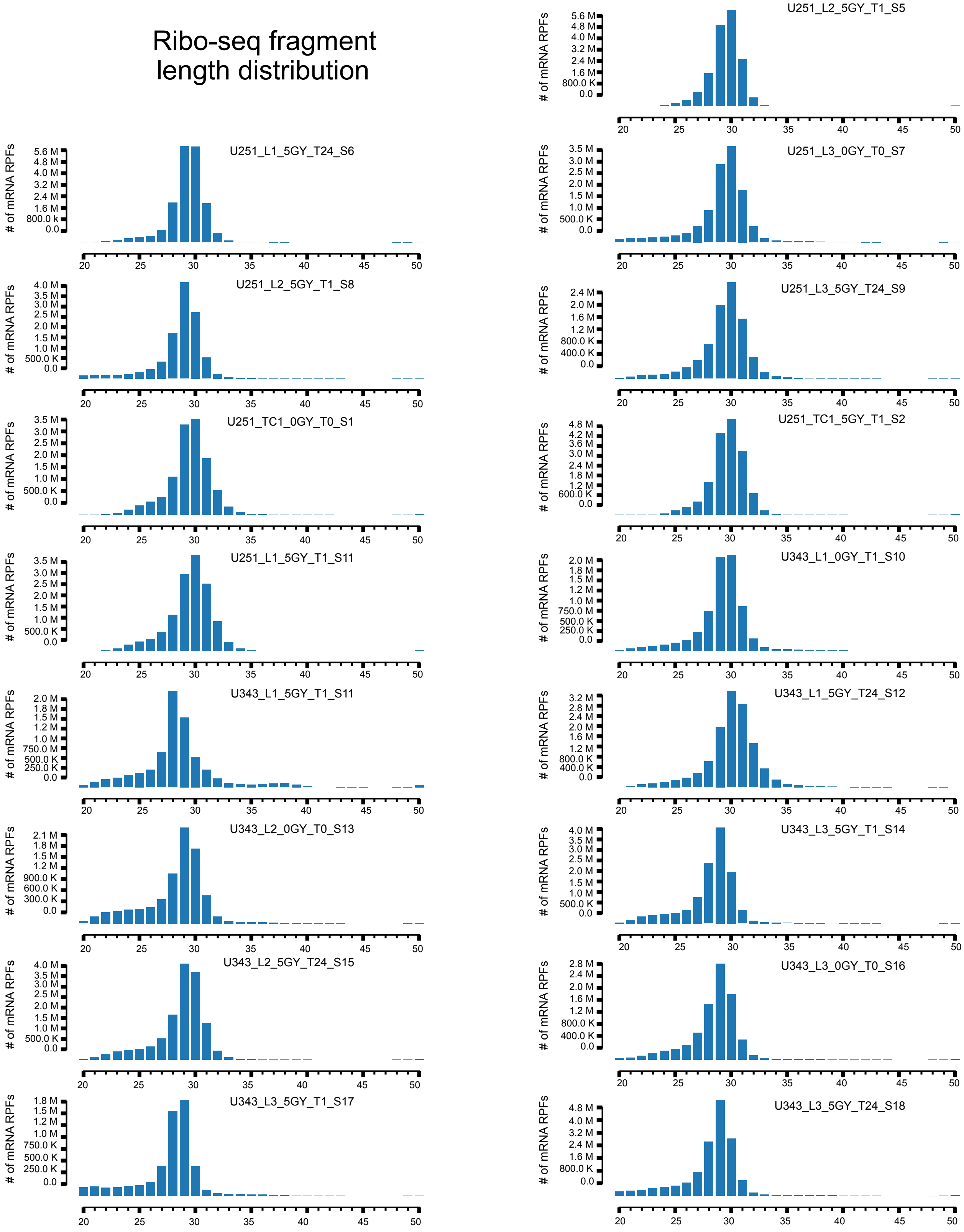

Figure S2: **Fragment length distribution of ribosome footprints of glioblastoma cell lines.** Fragment length distribution was obtained using ribotricer (v1.1.0).

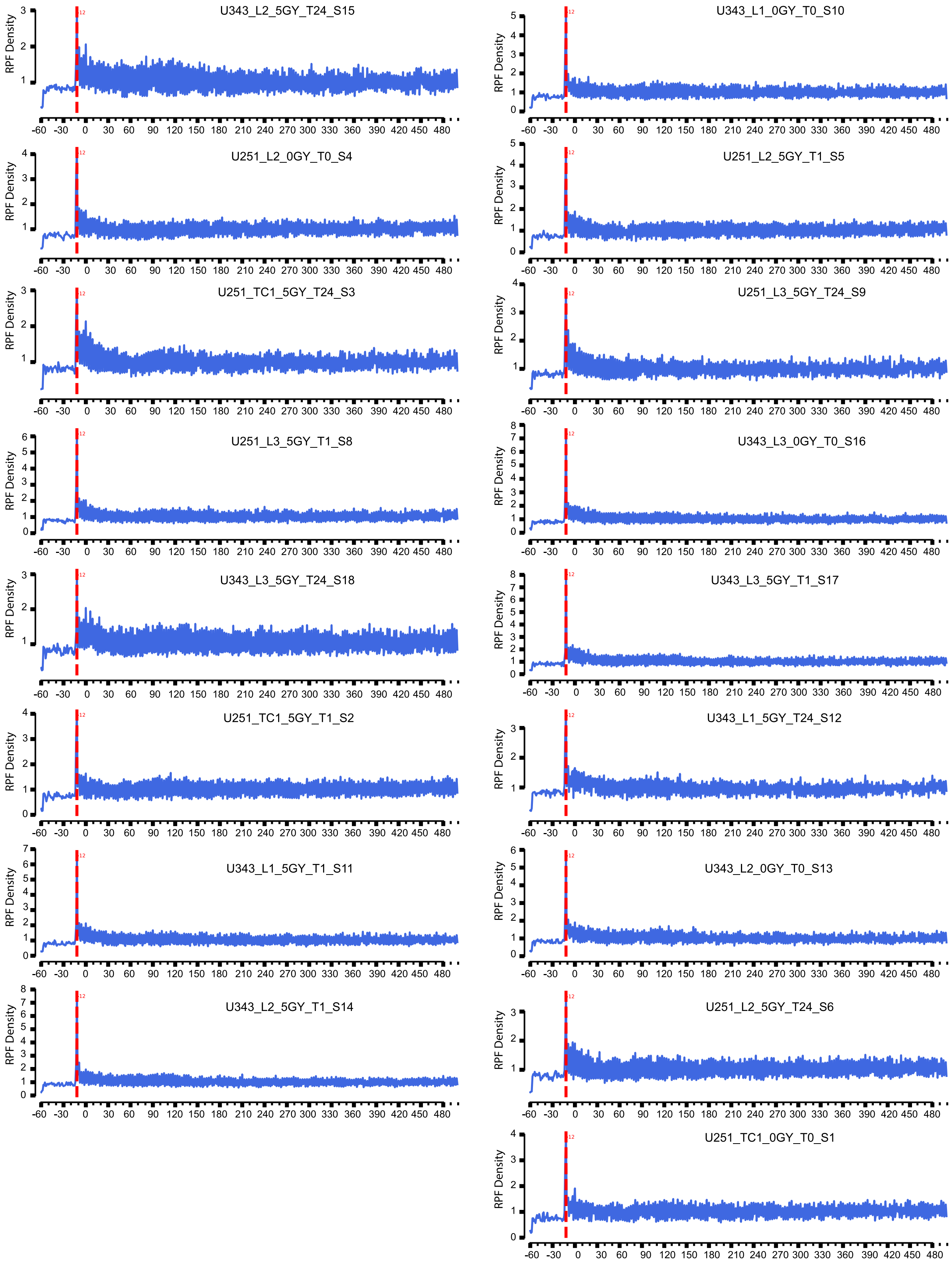

Figure S3: **The ribosome density profiles of glioblastoma cell lines.** Metagene distribution plots were obtained using ribotricer (v1.1.0).

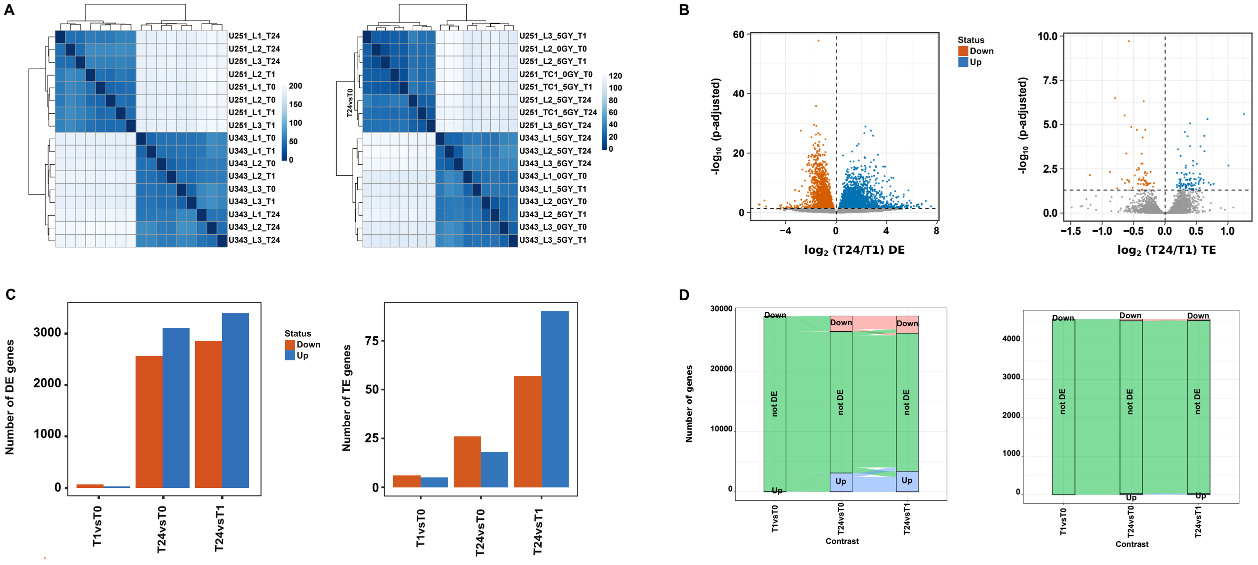

Figure S4: **A global view of glioblastoma cell lines transcription and translation profiles after radiation.** A) Heatmap indicating sample-wise distance on the logarithm scale of normalized read counts for RNA-seq (left) and Ribo-seq (right) samples. B) Volcano plots showing the expression (left) and translation alterations (right) of genes at 24 hr compared to 1 hr after radiation exposure. Blue dots indicate upregulated genes (adjusted p-value < 0.05, $\log_2$ fold change < 0), and orange dots indicate downregulated genes. (adjusted p-value < 0.05, $\log_2$ fold change < 0). C) Differentially expressed genes (left) and the number of genes whose translation efficiency is differentially regulated (right) after radiation exposure at different time points. D) Comparison of differentially expressed genes (left) and genes whose translation efficiency is differentially regulated (right) using the integrated (indicated as 'All') approach between three contrasts T1vsT0, T24vsT0, and T24vsT1.

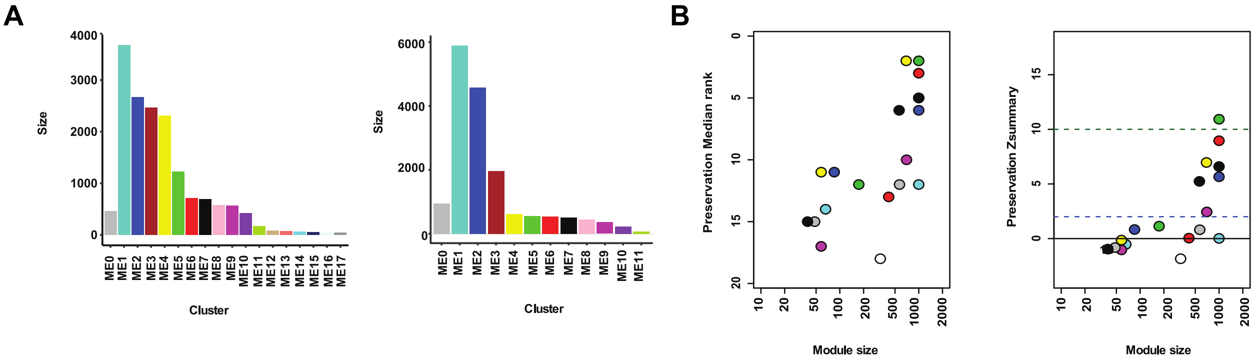

Figure S5: **Co-expression analysis of glioblastoma cell lines.** A) Sizes of gene modules found in U251 and U343 cell lines. B) Preservation Median Rank and Z_{summary} for all modules. A lower median rank indicates the module is preserved, and the corresponding modules in U251 and U343 cell lines share a high number of genes. A Z_{summary} score of 2-10 indicates weak preservation, while a Z_{summary} >10 indicates high preservation.

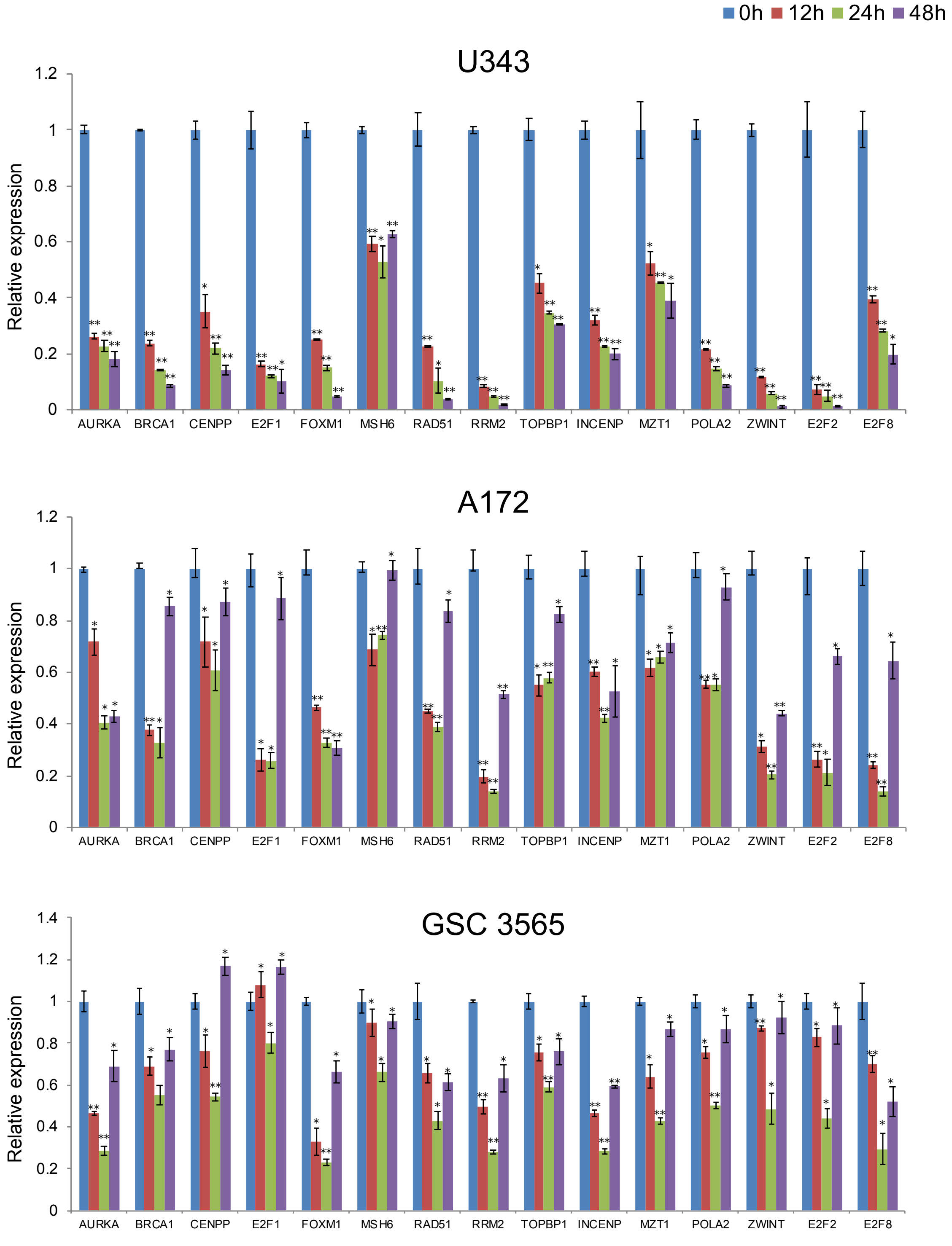

Figure S6: **Validation of changes in expression of DNA replication, DNA repair and cell cycle genes by qRT-PCR.** GBM cells U343 and A172 and GSC line 3565 were cultured till 80-90% confluent and then exposed to 5 Gy of ionizing radiation (IR), using a CP-160 Cabinet X-Radiator (Faxitron X-Ray Corp). Cells were collected at 12h, 24h and 48h after irradiation and changes in expression were analyzed by qRT-PCR. $p < 0.05*, p < 0.01 **$.
